# Metabolomic Dynamics Reveals Oxidative Stress in Spongy Tissue Disorder During Ripening of *Mangifera indica* L. Fruit

**DOI:** 10.3390/metabo9110255

**Published:** 2019-10-29

**Authors:** Pranjali Oak, Ashish Deshpande, Ashok Giri, Vidya Gupta

**Affiliations:** 1 Plant Molecular Biology Unit, Division of Biochemical Sciences, CSIR-National Chemical Laboratory, Pune 411008, India; 2 Academy of Scientific and Innovative Research (AcSIR), Ghaziabad 201002, India

**Keywords:** mango metabolomics, spongy tissue disorder, gamma amino butyric acid shunt, tricarboxylic acid cycle, oxidative stress

## Abstract

Spongy tissue disorder, a mesocarp specific malady, severely affects the flavor and pulp characters of Alphonso mango fruit reducing its consumer acceptability. Here, we investigated comparative metabolomic changes that occur during ripening in healthy and spongy tissue-affected fruits using high resolution mass spectrometric analysis. During the spongy tissue formation, 46 metabolites were identified to be differentially accumulated. These putative metabolites belong to various primary and secondary metabolic pathways potentially involved in maintaining the quality of the fruit. Analysis revealed metabolic variations in tricarboxylic acid cycle and gamma amino butyric acid shunt generating reactive oxygen species, which causes stressed conditions inside the mesocarp. Further, reduced levels of antioxidants and enzymes dissipating reactive oxygen species in mesocarp deteriorate the fruit physiology. This oxidative stress all along affects the level of amino acids, sugars and enzymes responsible for flavor generation in the fruit. Our results provide metabolic insights into spongy tissue development in ripening Alphonso mango fruit.

## 1. Introduction

Mango, the king of fruits, is well known for its unique flavor and high nutritional value. It is a source of energy, vitamins, essential minerals, proteins, carbohydrates, dietary fibers, etc. It is also rich in various phytochemicals such as quercetin, gallic acid, mangiferin, ascorbic acid, etc. which are the major antioxidants and essential for maintaining good health. The climacteric mango fruit has dynamic taste and is consumed at different stages of fruit development and ripening. India is the largest producer of mango and contributes to about 35% of the worldwide mango production (FAO, 2017). Globally, India ranks 7th in export of the mango, which plays an important role in earning foreign exchange for the country. Annually, around 50,000-ton mangos are being exported to various parts of the world including Middle East, Europe and the United States of America (FAOSTAT, 2016 (www.fao.org/faostat)). Though, India is the leading country in mango production, its cultivation is hampered because of climatic changes, variation in cultivation conditions and biotic stress [1]. Mango tree as well as fruit are susceptible to various diseases and disorders. Some common diseases in mango are powdery mildew, anthracnose, gummosis, mould, die back, etc. Disorders are the result of abiotic breakdown of tissue leading to change in fruit physiology [2]. They include imbalance in regular fruit metabolism caused by some pre-harvest (growth conditions, availability of water and nutrition) or post-harvest (temperature, oxygen, carbon dioxide level during storage) conditions. Identification of physiological disorder in fruit is difficult because of involvement of many abiotic stresses. These abiotic stresses alter the array of metabolic pathways and ultimately hamper the quality of fruit.

Mango fruit *per se* can be affected by various inherent and induced disorders at every stage of fruit development and ripening. Physiological disorders include black tip, malformation, fruit drop, spongy tissue, jelly seed formation, soft nose, stem end cavity, nutrient deficiency, etc. leading to changes in fruit dynamics that hamper the quality of fruit. Among all the disorders reported, spongy tissue disorder is the most devasting and difficult-to-detect as affected fruits do not show any external symptoms until fruits are cut opened, posing a challenge for quality control [3]. Spongy tissue disorder is characterized by mesocarp specific malady, wherein pulp around seed of the fruit shows white corky patches along with air pockets, keeping the affected part unripe [4].

Spongy tissue disorder has been reported in some Indian cultivars viz. Alphonso, Vanraj, Goamankur, Jamadar, Vellaikolamban, Olour, Swaranrekha and Fernandin and a few exotic cultivars, such as Tommy Atkins, Haden and Beverly. Totapuri, Sindhu, Ratna, Pairi, Kesar and Dasheri cultivars are resistant to spongy tissue formation [5]. Alphonso is the most popular cultivar because of its attractive color, the longest shelf life (15 to 20 days) and perfect blend of volatiles imparting highly attractive flavor to the ripe fruit [6]. Despite having the highest demand for Alphonso, its export as well as local market value is sometimes shackled because of this disorder. Almost 30% of the Alphonso mango export per year is reduced because of this malady [4].

Initial efforts to identify cause of spongy tissue disorder reported oxidative stress, early seed germination, cumulative heat and microbial hindrance [3,5,7]. Earlier, we reported higher accumulation of terpenes and green leafy volatiles, which is a peculiar characteristic of unripe mesocarp, while lower content of ripening specific lactones and furanones in spongy tissue [8]. Such change in volatile profile alters flavor of the fruit. Other studies also reported higher acidic content, lower carbohydrate metabolism, depleted mineral content in the spongy tissue, lowering nutritional value of the fruit [9]. This renders the fruit unfit for human consumption and fetches low value in the market. Various control measures, such as mulching of orchard, calcium dip treatment to fruit, hormonal treatments, etc. [9,10] have been suggested to minimize the agricultural loss, however, these measures only partially help in reducing the spongy tissue formation. As external physiology of the fruit does not change in this malady, studies reported so far have merely based on aftereffects of the disorder. Hence, in-depth biological knowledge regarding the inducing factors and the development of this difficult-to-identify-and-quantitate unpredicted disorder of the spongy tissue formation in mango is still very scanty and early detection is enigmatic to researchers.

Metabolomic approach has been widely used in certain fruits to elucidate various metabolites and metabolic pathways involved in peculiar characteristics of respective fruits. Temporal and spatial changes during the development and ripening of fruits such as mango, apples, banana, pear, cucumber and citrus [11,12,13,14,15,16,17] while pre- and post-harvest treatments to fruit in citrus [18] have been studied using metabolomic approach. Involvement of citric acid cycle in physiological disorder, viz. puffing disorder in citrus was also evaluated using in-fruit metabolomic approach [19].

Here, we performed microscopic imaging of mesocarp and biochemical studies such as targeted analysis of antioxidant capacity, phenolic and protein content and evaluation of three enzymes related to oxidative status of Alphonso mango fruit. To get detail insight into metabolic changes during spongy tissue formation, untargeted metabolomic analysis of the spongy tissue in mango was also carried out. Four ripening stages, viz. mature raw, table green, mid ripe and ripe stages of fruits with spongy tissue disorder were analyzed for stage specific changes in their metabolites and metabolic pathways. These were further compared to the respective stages of the spongy-control tissue from the same spongy fruit and completely healthy tissue of non-spongy fruit to identify metabolic dynamics responsible for spongy tissue formation in Alphonso mango fruit.

## 2. Results

### 2.1. Accumulation of Starch Granules and Intact Parenchyma Cells Characterize the Spongy Mesocarp in Mango

**Figure 1:**
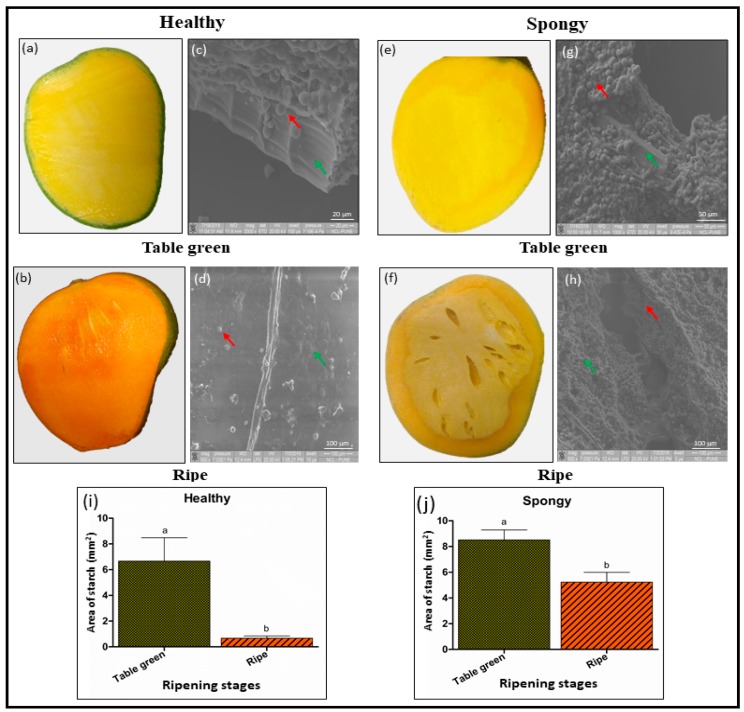
Cellular morphology of healthy and spongy mesocarp of mango fruit at table green and ripe stages. (**a**) and (**b**) represent the healthy mesocarp while and (**e**) and (**f**) represent spongy mesocarp at the above two stages. Representative scanning electron microscopic images of healthy (**c** and **d**) and spongy (**g** and **h**) mesocarp at two ripening stages are included adjacent to the respective fruit images. Red arrow indicates starch granules whereas green arrow indicates parenchyma cells. (**i**) and (**j**) histograms represent average area in mm^2^ covered with starch granules calculated from five different regions each of two respective SEM images of both the healthy and the spongy tissue, respectively at two stages. Normalization was done based on the scale represented on each image. Difference between ripening stage across tissue was significant (*p* ≤ 0.05) if lower case letters (a, b) above histogram are different.

Scanning electron microscopy of the table green and the ripe stage mesocarp of healthy and spongy tissue was performed to study spongy tissue specific anatomical changes in mango. These images showed uniform as well as high deposition of starch granules along with thick walled parenchyma cells (Figure 1a,c). At ripe stage, starch granules decreased significantly, and parenchyma cells were disrupted (Figure 1b,d). In contrast, in spongy tissue, the anatomy was much different with higher proportion of starch as compared to that in the healthy mesocarp (Figure 1e,g) at unripe stage. Moreover, accumulation of starch granules was persistent even in the ripe stage (Figure 1f,h). Quantitative image analysis also supported this observation (Figure 1i,j). In the healthy tissue, area under starch accumulation decreased 10-fold from unripe stage (6.6 ± 1.8 mm^2^) to ripe stage (0.6 ± 0.1 mm^2^) whereas in the spongy tissue though starch content significantly decreased (*p* ≤ 0.05) from unripe (8.5 ± 0.7 mm^2^) to ripe (5.2 ± 0.7 mm^2^) stage the fold change (1.6-fold) difference was less as compared to that in the healthy tissue.

Accumulation of starch granules even in the ripe stage suggested the delayed or arrested starch degradation and cell wall hydrolysis in spongy tissue disorder.

### 2.2. Altered Biochemical Composition of Fruit Mesocarp during Spongy Tissue Development

**Figure 2:**
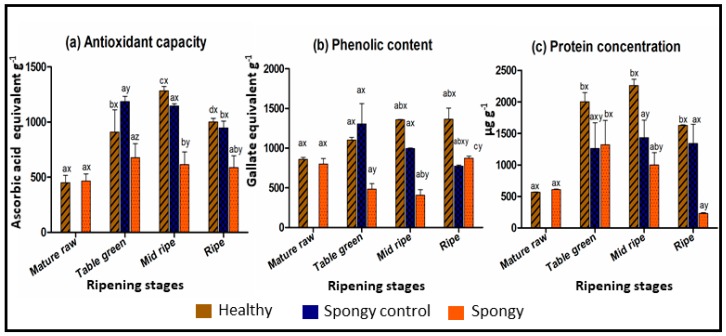
Biochemical assay. Average concentration of (**a**) antioxidant capacity in ascorbic acid equivalents per gram of tissue (AAE/g) (**b**) phenolic content in gallic acid equivalents per gram of tissue (GAE/g) and (**c**) protein concentration in µg/g of healthy, spongy control and spongy tissue mesocarp of Alphonso mango fruit at various ripening stages. Vertical bars represent standard error calculated from six biological replicates. Difference between the stages of each tissue set was significant (*p* ≤ 0.05) if lower case letters (a, b, c) are different. Difference between the tissue sets at each ripening stage was significant (*p* ≤ 0.05) if lower case letters (x, y, z) are different.

Change in the mesocarp anatomy of spongy tissue is an indication of biochemical dynamics in the fruit because of the disorder development. Important biomolecules such as proteins, phenolics and antioxidants were, therefore, assayed in healthy, spongy-control and spongy tissue at all the four stages of ripening. An antioxidant capacity ranged from 455 ± 66 aae/g to 1283 ± 39 aae/g in healthy and spongy-control tissue, while that of spongy tissue was in the range of 466 ± 64 to 677 ± 127 aae/g during ripening. It was significantly less in spongy tissue mesocarp as compared to that in the other two tissue sets at each stage of ripening (Figure 2a). The phenolic content increased in the healthy (1363 ± 140 gae/g) while increased initially and decreased at later stages in the spongy-control tissue during ripening (774 ± 13 gae/g). However, in case of spongy tissue it decreased in the initial stages and increased at the ripened stage (873 ± 25 gae/g). However, the content was significantly less in spongy tissue as compared to that in the healthy mesocarp at all the stages of ripening (Figure 2b). Healthy and spongy-control tissue showed positive correlation between antioxidant and phenolic content through all the ripening stages (0.84 and 0.80, respectively) whereas the correlation was negative in spongy tissue (−0.23). This indicated that the spongy tissue possessed higher load of phenolics than antioxidants as compared to that in the other tissue sets.

In the healthy and the spongy-control tissue, protein concentration increased till mid ripe stage and remained higher at ripe stage. However, in the spongy mesocarp, protein content decreased significantly from the table green (1.3 ± 0.3 mg/g) to the mid ripe stage (0.9 ± 0.1 mg/g) and was minimum at the completely ripe stage (0.2 ± 0.01 mg/g) as compared to that in the other tissue sets (Figure 2c). Such lower concentration at mid ripe and ripe stages indicated lower protein synthesis or higher protein degradation, probably affecting the enzyme activity in the spongy mesocarp.

**Figure 3:**
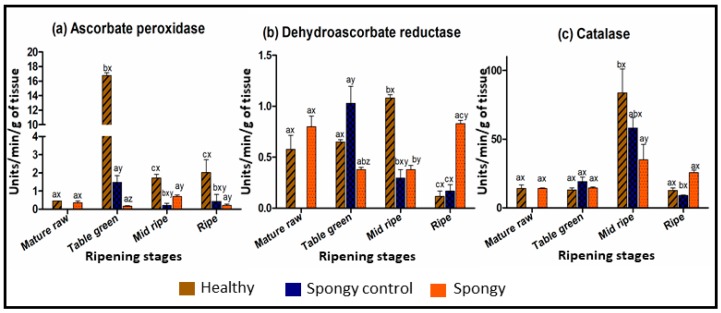
Enzyme assay. Average enzyme activity of (**a**) Ascorbate peroxidase (**b**) Dehydroascorbate reductase and (**c**) Catalase in units/min/g of healthy, spongy control and spongy tissue mesocarp of Alphonso mango fruit at various ripening stages. Vertical bar represents standard error calculated from six biological replicates. Difference between the stages of each tissue set was significant (*p* ≤ 0.05) if lower case letters (a, b, c) are different. Difference between tissue sets at each ripening stage was significant (*p* ≤ 0.05) if lower case letters (x, y, z) are different.

Lower antioxidant capacity and protein content in spongy mesocarp has further directed the comparative analysis of antioxidant related enzyme activities. Ascorbate peroxidase activity was very high at the table green stage as compared to other stages in the healthy tissue (16 ± 0.3 units min^−1^ gm^−1^) whereas it was very low for the spongy-control (1.4 ± 0.3 units min^−1^ gm^−1^) and spongy tissue (0.1 ± 0.03 units min^−1^ gm^−1^) both at all the stages of ripening (Figure 3a). Activity of dehydroascorbate reductase was significantly low in the spongy tissue (0.3 ± 0.02 units min^−1^ gm^−1^) as compared to the healthy tissue at the table green (0.6 ± 0.02 units min^−1^ gm^−1^) and the mid ripe (1.08 ± 0.03 units min^−1^ gm^−1^) stage but at the ripe stage enzyme activity was significantly higher in the spongy tissue (0.8 ± 0.03 units min^−1^ gm^−1^) as compared to that in the other two tissue sets (0.1 ± 0.05 units min^−1^ gm^−1^) (Figure 3b). Catalase activity was maximum at the mid ripe stage for all the three tissues. Though similar pattern was observed for all the tissues, at the mid ripe stage catalase activity was minimum for the spongy tissue (35 ± 11 units min^−1^ gm^−1^) followed by the spongy-control tissue (58 ± 7 units min^−1^ gm^−1^) while it was maximum for the healthy tissue (83 ± 17 units min^−1^ gm^−1^) (Figure 3c).

### 2.3. Plethora of Metabolites Involved in Spongy Tissue Formation

Untargeted metabolic profiling of all the three tissue sets at four ripening stages was performed to understand the variations in metabolites, if any, during spongy tissue formation in Alphonso mango using high resolution mass spectrometric analysis with liquid chromatography.

The stability and reproducibility of the LC-MS instrument were confirmed by running the QC sample. Stability was checked by retention time, mass accuracy and peak intensities of selected EIC’s (leucine and isoleucine) from QC samples. Mass accuracy of 1 ppm was considered during data acquisition. Coefficient of variation for leucine and isoleucine intensities were 11.4% and 14.9%, respectively and alignment of EIC’s suggested least deviation in retention time (Figure S1) calculated from total eight individual QC runs.

**Figure 4:**
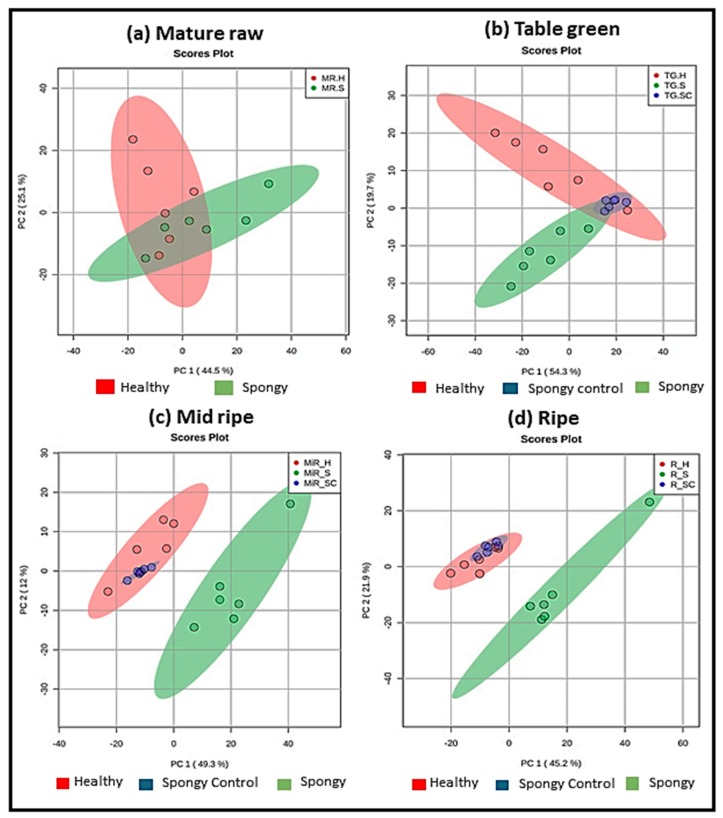
Stage specific metabolite feature variation. PCA Score plot using metaboAnalyst 4.0 distinguishing three tissue sets; healthy: H, spongy control: SC, spongy: S through four ripening stages (**a**) mature raw: MR, (**b**) table green: TG, (**c**) mid ripe: MiR, (**d**) ripe: R of mesocarp of mango. Total 10233 features from six biological replicates for each tissue set were considered.

Metabolic profiling of all the three tissue types led to identification of 10233 features under all the experimental conditions (Table S1). Out of these, 3046 features were successfully annotated, whereas, identity of 7187 features could not be assigned based on KEGG database. Multivariate and univariate analysis was further performed to select the features that were differentially expressed within three tissue sets (Figure S2). PCA was carried out separately for each stage of ripening across tissue types (Figure 4a–d) as well as for each tissue type separately across all the ripening stages (Figure S3). Ripening stage specific PCA (Figure 4a–c) showed that at the mature raw stage there was no separation of metabolite features within healthy and spongy mesocarp. It started from the table green stage with complete separation in the mid ripe and the ripe stage. Interesting observation was in case of the spongy and the spongy-control tissues, though they belonged to the same fruit, the sample separation was evident from table green stage till the ripe stage. In all the analyzed stages of ripening, samples from the healthy and the spongy-control tissue grouped together suggesting no change in metabolite feature profile among these two sets. This can be supported visually from similar mesocarp morphology of these two tissues as compared to that of the spongy mesocarp. In tissue specific variation (Figure S3a–c), the first two dimensions of PCA contributed the highest in the healthy tissue (57%), intermediate for the spongy-control (50%) and the lowest for the spongy tissue (43%). In the healthy tissue there were some overlaps in the metabolite features in the ripening stages with clear separation at the ripe stage. Whereas, in the spongy and the spongy-control tissues there was complete demarcation of the features at the ripe stage. A two-way ANOVA (analysis of variance) (FDR ≤ 0.05) was then carried out to understand which factor (ripening stage and/or tissue type and/or their interaction) caused the variation in each metabolite feature (Figure S4). Among 10,233 features detected, 5135 features were simultaneously affected by all the three factors. Abundance of 51, 252 and 50 features revealed variation by tissue phenotype, stage of ripening and their interaction, respectively. These results suggest that, maximum change in metabolite feature profiling occurred during ripening of fruit.

**Figure 5:**
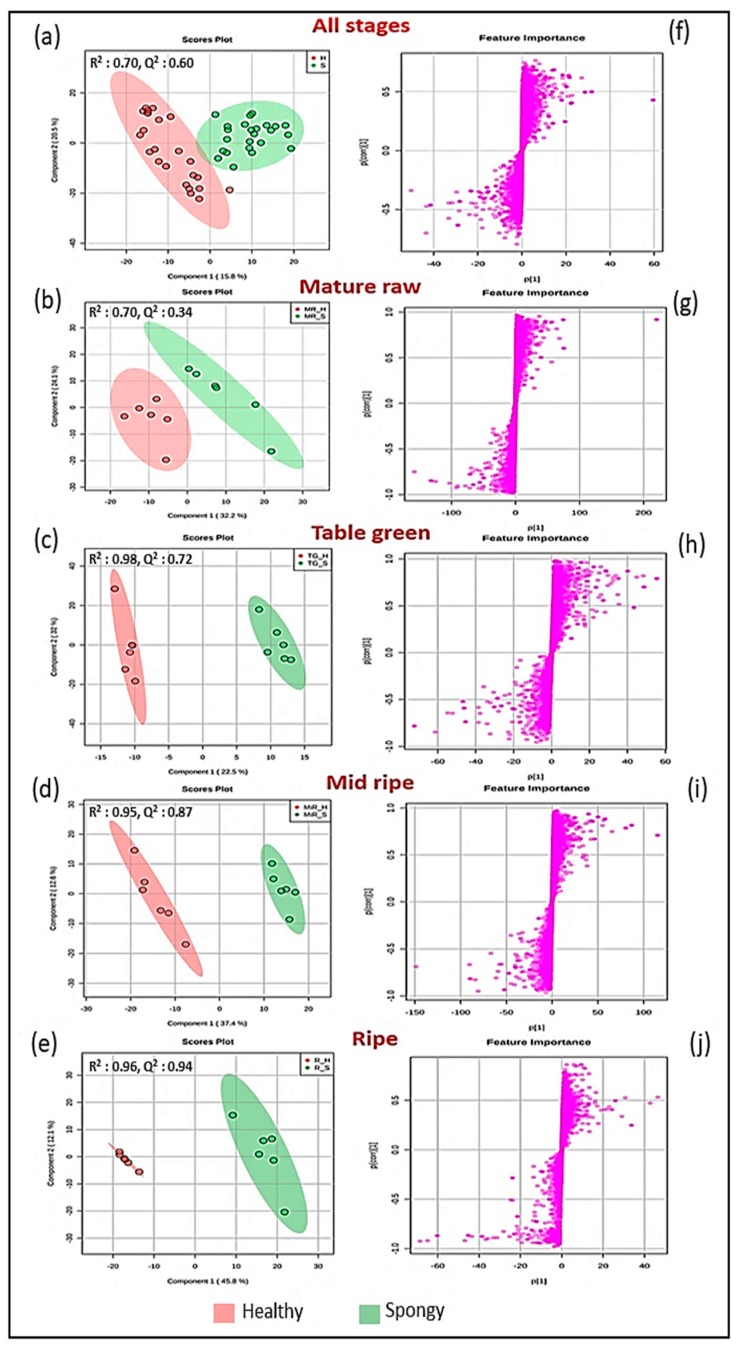
Pairwise metabolite feature comparison. PLS-DA Score plot (**a** to **e**) and corresponding S-Plot (**f** to **j**) obtained from metabolic profile considering healthy and spongy tissue at all the stages combined and four ripening stages (mature raw, table green, mid ripe and ripe) individually of the mesocarp of mango.

Partial least square-discriminate (PLS-DA) analysis between the healthy and the spongy tissues at all the ripening stages revealed a complete separation between the healthy and the spongy tissue at each time point and at all the stages together with distinguished clusters (Figure 5a–e). Moreover, CV-ANOVA (cross validation analysis of variance) validated model parameters in the permutation test (2000 permutation numbers) for the explained variation (*R*^2^) and the predictive capability (Q^2^), which were significantly high (>0.5). This indicated the excellence in modelling and prediction with good discrimination among the healthy and the spongy mesocarp. Post processing OPLS-DA analysis revealed covariance and correlation between the metabolite variables and the model. Potential metabolites among thousands of variables from the healthy and the spongy fruit were determined based on the S-plots (Figure 5f–j), which preferentially showed a high covariance combined with a high correlation. The effect of every variable on the classification was reflected from their loading and variable importance in projection (VIP) value. Metabolic variables with high influence on loading and VIP ≥ 2 were taken for further analysis. To further remove the features with low reliability, only the biologically important variables with the significant difference in peak intensity variation (*p* ≤ 0.05) within the three tissue sets were identified. Thus, MS/MS analysis of 285 metabolites was performed in positive as well as negative mode.

### 2.4. Metabolic Alterations in Spongy Tissue Formation

Based on multivariate statistical analysis (VIP ≥ 2, S-Plot), fragment matching after MS/MS analysis (minimum 6), metlin score (minimum 80), retention time, ion annotation, peak intensity calculation (univariate analysis with *p* ≤ 0.05) and biological relevance, a total of 46 metabolites were putatively confirmed at least up to level 2 (Table 1).

These putative metabolites included amino acids, sugars, glucosides, fatty acid derivatives, vitamins, flavonoids, compounds involved in various primary and secondary metabolic pathways such as tricarboxylic acid cycle, pentose sugar phosphate cycle, fatty acid synthesis, etc.

### 2.5. Profiling of Metabolites Involved in Primary Metabolism

Differential pattern of the putative metabolites is depicted based on the peak intensities of each metabolite in three tissue sets at all the stages of ripening. Amino acids such as leucine, lysine, methionine, tyrosine, tryptophan, threonine and valine were observed in ripe stage of the spongy tissue as compared to the healthy and the spongy-control mesocarp (Figure S5a).

**Figure 6:**
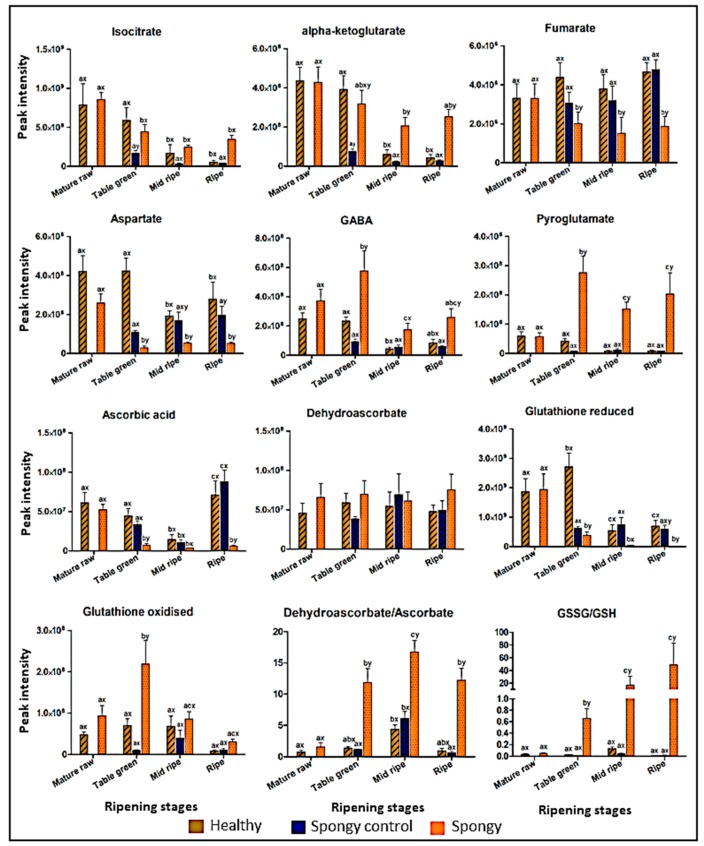
Metabolites involved in TCA Cycle, GABA shunt and Ascorbate glutathione cycle. Differential accumulation of metabolites in healthy, spongy control and spongy tissue within four stages of ripening (mature raw, table green, mid ripe and ripe) of Alphonso mango. Y-axis denotes the peak intensity values. Vertical bars represent standard error within six biological replicates. Difference between the stages of each tissue set was significant (*p* ≤ 0.05) if lower case letters (a, b, c) are different. Difference between tissue sets at each ripening stage was significant (*p* ≤ 0.05) if lower case letters (x, y, z) are different.

Phenylalanine and arginine content decreased during ripening in all the tissue sets but the abundance was found to be significantly higher in the spongy tissue as compared to the spongy-control and healthy tissues. On the contrary level of histidine which is an important amino acid involved in proper ripening of a fruit was significantly high at ripe stage of the healthy and the spongy-control tissue as compared to the spongy mesocarp (Figure S5a,b). Intermediate and key metabolites involved in tricarboxylic acid cycle (TCA), alpha ketoglutaric acid and isocitrate, consistently decreased during ripening in the healthy and the spongy-control tissue (Figure 6). Similar trend was observed in the spongy tissue except that the levels were significantly higher in the ripe stage as compared to that in the intermediate stages while concentration of alpha ketoglutaric acid in spongy tissue was significantly higher in all the stages of ripening as compared to that in the healthy and the spongy-control mesocarp, except at the mature raw stage where the values were similar. Further in the pathway, fumarate and aspartate was found to be significantly lower in the spongy tissue. Surprisingly, well known anaerobic stress signature molecule, gamma amino butyric acid (GABA) [21] and pyroglutamate (precursor of glutamate, a substrate for GABA synthesis) was accumulated higher in the spongy tissue as compared to the other two tissue sets at all the stages with maximum at the table green stage.

Like in the TCA cycle, metabolites involved in ascorbate glutathione cycle showed differential accumulation patterns. Current metabolite extraction protocol was not specific to preserve these metabolites and their recovery rate after extraction was not checked. However, the significant accumulation (*p* ≤ 0.05) in spongy tissue was depicted based on the respective peak intensity values in the three tissue sets. The concentration of reduced forms of ascorbic acid and glutathione, important compounds in coping up with oxidative stress, was found to be significantly lowered in most of the stages in the spongy tissue as compared to the healthy mesocarp. While that of oxidized glutathione and dehydroascorbate depicted higher concentration in the spongy tissue as compared to that in the other two tissues at most of the stages, Interestingly, the ratio of concentration of oxidized to reduced forms was significantly higher in the spongy tissue as compared to the healthy and the spongy-control tissue at all the ripening stages shifting the redox balance in the fruit (Figure 6).

The concentration of sedoheptulose, an intermediate in pentose phosphate pathway increased at all the stages of ripening in all the tissue sets but its concentration in the spongy-control tissue was significantly higher in all the stages (Table 2 and Figure S5b). In the spongy mesocarp, level of vitamin B_6_ glucoside (5’-O-β-D-glucosylpyridoxine), which has been recently reported to be involved in managing the stress conditions in plants [22] and contributing up to 75% of vitamin B_6_ budget [23] increased in the table green (7-fold) and the mid ripe (27-fold) stage and was observed maximum at the ripe stage (92-fold) as compared to the mature raw stage of the healthy tissue (Table 2 and Figure S5b).

### 2.6. Variation in the Flavor Metabolites in Spongy Tissue

**Figure 7:**
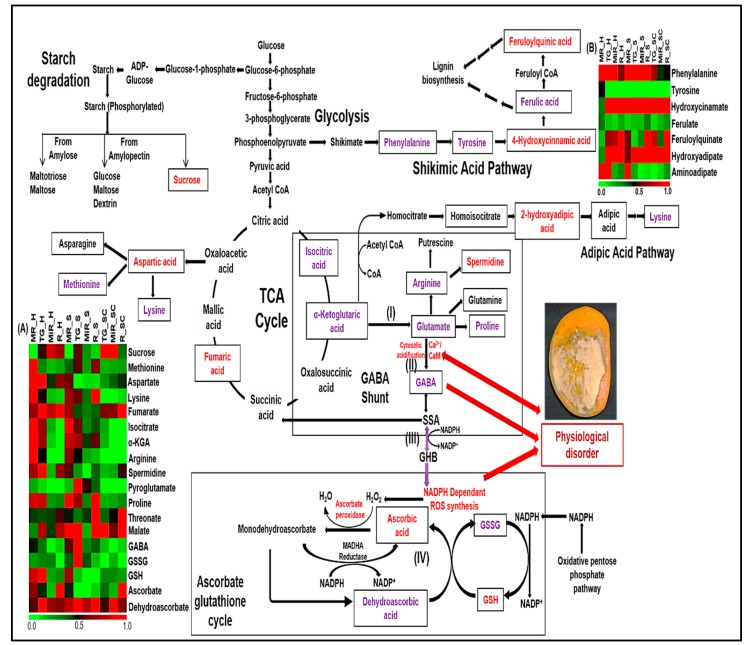
Probable pathways involved in spongy tissue formation. Ripening stage wise accumulation of each metabolite in all the three tissue sets is represented by heat map (MR: Mature raw, TG: Table green, MiR: Mid ripe, R: Ripe, H: Healthy, S: Spongy, SC: Spongy control). Heatmap (**A**) represents the putative metabolites from starch degradation, amino acid synthesis, TCA and ascorbate glutathione cycle whereas Heatmap (**B**) represents the metabolites from shikimic acid and adipic acid pathway. Metabolites identified in analysis are separated by box. Metabolites which are upregulated at the completely ripe stage of spongy tissue as compared to the healthy and the spongy control are represented in blue whereas downregulated metabolites are highlighted in red. Other metabolites are the components of respective pathways but are not identified in the current analysis. Black arrow indicates normal metabolic pathways whereas blue arrow (SSA to ROS synthesis) represents the probable metabolic variation which may induce physiological disorder. Pathways in which probable metabolic change occurs (GABA shunt and Ascorbate glutathione cycle) are highlighted separately. (Figure modified from [25]).

Flavor-related metabolites were found to be significantly less (*p* ≤ 0.05) in the spongy tissue as compared to the healthy and the spongy-control tissues during ripening. 1-Aminocyclopropane [24], a precursor of ethylene (ripening hormone), gradually increased from the table green stage to ripe stage in the healthy and the spongy-control tissue whereas these levels were significantly lower in the spongy tissue during ripening probably hampering the ethylene production in the spongy tissue. The pattern of abscisic acid (ABA), which is important in the process of fruit ripening, was also studied. During ripening, level of ABA in the healthy and the spongy-control tissues increased from the mature raw stage to the ripe stage in the range of 2.83 to 4.12 and 1.54 to 2.23-fold, respectively. However, in case of the spongy tissue, level of ABA was significantly less at each stage of ripening and the fold change was only in the range of 0.35 to 1.21 (Table 2). Sucrose and metabolites involved in adipic acid and sinapinic acid biosynthesis such as 2-hydroxyadipic acid, hydroxycinnamic acids were found to be less in the spongy tissue as compared to the healthy tissue set (Table 2). Level of Feruloylquinic acid and coronarian, which are the intermediate metabolites in shikimate or phenylpropanoid pathway were less in the spongy tissue. However, the levels of phenolics such as ferulic acid, chlorogenic acid, naringenin and coniferin increased gradually in the spongy tissue during ripening. As like phenolics, some glucosides were also found to be increased during ripening (Figure 7, Table 1, Figure S3). Deideclein, a cynogenic glucoside and ranunculin, an unstable glucoside which is converted to glucose and toxin protoanemonin (upon wounding), was significantly accumulated in the spongy mesocarp at the mid ripe and the ripe stage. A sesquiterpene lactone, vernoflexuoside showed higher fold change in the spongy tissue during ripening (24-fold) (Table 2). We also observed a higher accumulation of the hydroxy fatty acid 9,10-dihydroxy-12,13-epoxyoctadecanoic acid in the mid ripe and the ripe stage of the spongy tissue. It was 36 and 133-fold more, respectively, as compared to that in the mature raw stage of healthy tissue. Whereas, in the healthy and the spongy-control tissue increase was only up to one- or two-folds during the ripening process (Table 2, Figure S5).

Thus, in case of the spongy tissue of mango there were phenotypic and biochemical changes in the mesocarp and abundance of stress related metabolites, whereas significant reduction in the metabolites contributing to flavor of the ripe mango.

## 3. Discussion

Fruits are the major nutritional sinks of plants. Fruit physiology and fruit ripening are the complex processes involving various biological interactions between biomolecules such as nucleic acids, proteins, carbohydrates and lipids. Any alteration in these interactions leads to change in the physiology of the fruit. Metabolomics approach provides a comprehensive study of these cellular metabolites, thus representing the absolute physiological state of a cell [13]. It has been widely used to study the metabolic changes in fruits such as tomato [26], citrus [19], pear [15], cucumber [16] and pepper [27] under various ripening and stress conditions. It generates enormous information of differential metabolites, which helps in identification of a probable biomarker. Hence metabolomics approach has been used to get insight into the changing dynamics of metabolites and their pathways which may provide leads for spongy tissue formation in mango. The metabolites identified in the present study are at level 2 of MSI identification and are putative in nature, however, their significant role in understanding abiotic stress-based malady in mango as discussed below.

### 3.1. Cellular Changes in Spongy Mesocarp Hamper the Internal Physiology of Fruit

During the process of fruit ripening, thick parenchyma cells of mango mesocarp becomes thin and the embedded starch granules subsequently disappear because of their hydrolysis [28]. Scanning electron microscopy of ripening stages of the healthy mesocarp in mango clearly demonstrated the thick parenchyma cells and starch accumulation at the mature raw stage while smudging of starch granules and thinning of parenchyma cell wall in the ripe tissue. However, in case of spongy tissue mesocarp, accumulation of starch granules and intact parenchyma cells were persistent even at the ripe stage. Due to higher starch accumulation, texture and taste of the mesocarp changes, thus making the fruit unfit for consumption. Such textural changes were probably due to lower activity of starch hydrolyzing and cell wall degrading enzymes which hampers the process of fruit ripening (Figure S6). Lower activity of ripening related enzymes has been reported in spongy tissue disorder [8,29].

### 3.2. Deviation in Primry Metabolism in Spongy Tissue Disorder Highlights the Stress Conditions in the Fruit

Various studies have been carried out to fetch a probable cause behind spongy tissue formation, and the highlighted reasons are accumulation of heat, osmotic gradient formation in seed and mesocarp, higher respiration rate in mesocarp, etc. [30,31]. It is well known that adverse changes in physiological conditions of fruits affect the biomolecules, such as ripening induced enzymes, carbohydrates, amino acids, etc. present in the mesocarp, which ultimately modulates the metabolic process in the fruit. In the current study, metabolites involved in respiratory pathways such as TCA have shown differential pattern in the spongy tissue as compared to the healthy one. Level of metabolite alpha ketoglutarate was higher while fumarate and aspartate contents were significantly less in the spongy tissue set. Intermediate metabolites such as succinic acid, malic acid and oxaloacetic acid were not detected in the current analysis. These three observations in the spongy tissue as compared to that in the healthy and the spongy-control in the present study suggests the diversion of TCA cycle from alpha ketoglutarate in spongy mesocarp (Figure 7I). α-Ketoglutarate, a derivative of glutaric acid, acts as a precursor for the synthesis of an amino acid, glutamine via glutamate formation where pyroglutamate also plays an important role in the process. Glutamate also acts as a substrate for synthesis of GABA. In case of the spongy tissue, levels of pyroglutamate and GABA were higher in the table green stage than the mature raw stage of ripening followed by the ripe and the mid ripe stages (Figure 6). Overall concentration of these compounds in the spongy tissue was also significantly higher at all the stages of ripening as compared to that in the healthy and the spongy-control tissue. Thus, it confirms the diversion of TCA cycle from α-Ketoglutarate to the glutamine biosynthesis in the spongy tissue (Figure 7II). It has been well studied that in tomato fruits level of GABA is maximum in immature fruit and decreases during fruit ripening [21,32]. We observed similar trend in the healthy and the spongy-control tissue while opposite in the spongy tissue of Alphonso as detailed above.

Usually, abiotic stress conditions result in higher accumulation of NADH which inhibits the activity of succinic semialdehyde dehydrogenase and activates succinic semialdehyde reductase. This enzyme diverts the pool of succinic acid formation to gamma hydroxy butyrate (GHB) and stalls the TCA cycle [25]. Interestingly, succinic acid and further metabolites in TCA cycle were either in low amount and/or not detectable in the spongy tissue. Thus, the block in the GABA shunt can be hypothesized along with the formation of GHB or other GABA derivatives (Figure 7III) in the spongy tissue. GHB triggers the NADPH dependent ROS synthesis leading to the accumulation of peroxides and oxides which damage the cellular structure of mesocarp. Microscopic analysis of spongy mesocarp precisely highlighted the cellular disintegration, probably leading to cytosolic acidification. This is responsible for GABA accumulation which also leads to down regulation of cell wall modification genes involved in development and ripening of a fruit [33]. Higher accumulation of GABA and its derivatives has been reported in abiotic stresses such as chilling, low O_2_, elevated CO_2_ and mechanical stimulation in plants., that ultimately leads to physiological disorders in fruits [34]. GABA along with plant arginine decarboxylase enzyme also acted as a modulator leading to hydrogen peroxide burst in fruits of soybean under biotic stress conditions [27,35]. Such increased redox burst has also been partially reported in spongy mesocarp [10,36]. Thus, the reduced rate of catabolism of GABA and/or increased flux in the direction of GABA shunt than TCA cycle affects the plant resilience and development [37]. Because of ROS synthesis and metabolic fluctuations, synthesis of certain amino acids (product of TCA cycle) was seen to be significantly hampered in the spongy tissue. Hence, accumulation of GABA and its derivatives but reduced levels of amino acids highlight the extreme stress conditions inside the mesocarp of the spongy tissue in mango.

Sedoheptulose, which plays a vital role in non-oxidative branch of pentose phosphate pathway, was found to be accumulated in the spongy-control tissue. The sub-cellular localization and precise role of sedoheptulose in C3 plants is unknown. It has been proposed in Crassulacean Acid Metabolism [26] plants that free sedoheptulose contributes to both, carbon and phosphorus homoeostasis in the leaves of *Kalanchoe pinnata* plants by acting as an alternative carbon store under elevated CO_2_ level to avoid sucrose accumulation and depletion of inorganic phosphates [38]. In case of the spongy tissue, elevated levels of CO_2_ have been reported [36] which might have triggered the synthesis of sedoheptulose in surrounding tissue i.e., the spongy-control tissue.

### 3.3. Abiotic Stress Induces Cellular Oxidative Damage Leading to Spongy Tissue Formation in Alphonso

Mango being a climacteric fruit, balance between rate of transpiration and respiration plays an important role in ripening. Earlier, studies have been carried out to understand the mechanism of respiration in the mango fruit. The lower transpiration rate in Alphonso might be the ‘varietal specific trait’ leading to slower movement of water and minerals, while higher rate of respiration probably leads to accumulation of heat and CO_2_ inside the fruit [7,39]. This affects cellular structure of the mesocarp and causes acidic and anaerobic condition as well as ROS production inside the fruit. In the present study, lower content of total antioxidants in the spongy tissue suggested decreased ROS scavenging activity. This was accompanied with lower activity of ROS dissipating enzymes, catalase, dehydroascorbate reductase and ascorbate peroxidase in the spongy tissue as compared to the healthy one. Membrane peroxidation is the hallmark for detection of ROS generation and accumulation [40]. Fatty acid analysis in the spongy tissue disorder in previous studies has shown higher ratio of linoleic to linolenic acid, which indicates the peroxidation of membrane lipids [8,41]. Such ROS mediated membrane disintegration has also been reported in flesh browning disorder in apple *Cv.* ‘pink lady’ mesocarp [14].

Macro- and micro-mineral analysis of spongy tissue mesocarp has shown higher concentration of magnesium, zinc and iron but decreased concentration of calcium, sodium and potassium [42]. Such decreased calcium content and cytosolic acidification along with ROS generation, leads to a shift in reduced to oxidized forms of nicotinamide adenine dinucleotide phosphate (NADPH/NADP^+^) cofactors [25]. NADPH also plays an important role in ascorbate-glutathione recycling pathway (Figure 7IV). Ascorbate and glutathione are oxidized by ROS. Net result of ascorbate-glutathione cycle is the regeneration of reduced forms of ascorbate and glutathione while reducing the ROS in the form of alcohol or water. It is a coordinated interaction of glutathione reductase and ascorbate peroxidase enzymes. It has been studied that under low oxygen conditions, dehydroascorbate and glutathione disulphide tend to accumulate due to decline in the rate of interconversion in their reduced forms [40] which might be because of NADPH utilization in ROS generation [43]. Metabolic analysis of the spongy-control tissue in the current study showed higher level of ascorbic acid. Similar conditions have been seen in core breakdown disorder in the mesocarp of pear which indicates anoxia inside the fruit [44]. In the present analysis of completely developed spongy tissue, lower level of ascorbic acid while abundance of oxidized forms of ascorbic acid and glutathione, viz. dehydroascorbic acid and oxidized glutathione, were observed. Similarly, higher level of threonate which is a product of ascorbic acid catabolism [45] was also revealed in spongy tissue. Such higher accumulation of threonate indicates the dysfunction of ascorbate-glutathione cycle which is most likely due to the depletion of cellular reducing equivalents (NADPH) required to regenerate glutathione for dehydroascorbate reductase mediated conversion of dehydroascorbic acid to ascorbic acid. Reduced ascorbate peroxidase activity leads to the accumulation of peroxides which also contributes to the increasing stress conditions inside the fruit. Increased abiotic stress in mesocarp leads to deactivation of enzymes involved in various metabolic processes. Higher concentration of certain amino acids, lower protein content and reduced enzyme profiles observed in the spongy tissue mesocarp in the present study, highlighted the stress conditions in the mango fruit with spongy tissue disorder.

Higher accumulation of vitamin B_6_ (pyridoxal phosphate) has been correlated with abiotic stress conditions in plants [22]. In the spongy tissue, sudden increase in vitamin concentration was observed at the mid ripe and the ripe stages which indicated the onset of stress conditions and spongy development from the table green stage of ripening. Stress condition in the spongy tissue was also supplemented from the higher accumulation of phytosphingosine than that of sphinganine (Figure S5a). In the healthy tissue, level of sphinganine and phytosphingosine gradually decreased during ripening. On the contrary, levels of phytosphingosines were significantly higher in the spongy tissue as compared to the healthy and the spongy-control tissue at all the stages of ripening. Significantly low levels of sphinganine in the spongy mesocarp probably indicated its conversion into phytosphingosine. It has been reported in *Arabidopsis thaliana* that *Pseudomonas* infection in the leaves triggered *de novo* synthesis of phytosphingosine from sphinganine [46]. Along with biotic stress, abiotic stress such as cold shock and drought conditions also induced the production of phytosphingosine in plants [47].

### 3.4. Modulation in Secondary Metabolites Affects the Flavor of Fruit in the Spongy Tissue Disorder

Ethylene plays an important role in post-harvest fruit ripening and triggers the metabolic pathways leading to changes in taste, color and texture of the fruit. In the present study, amino cyclopropane carboxylate [24], which is a precursor of ethylene was found to be significantly less in the spongy tissue mesocarp during the fruit ripening whereas converse was observed in the healthy and the spongy-control mesocarp, which was contradictory to the earlier report [36]. More ACC content in the spongy tissue (0.34 µg/g of fresh weight) as compared to the healthy tissue (0.12 µg/g of fresh weight) might be due to the variation in detection method and discrepancy in the ripening stages. The low concentration of ACC content leading to reduced conversion to ethylene might have hampered the ripening process by arresting the mesocarp in the unripe stage in the spongy tissue as observed in the present study. Plant hormone abscisic acid also plays an important role in ripening of fruit by influencing fruit softening, aroma and anthocyanin biosynthesis [48,49]. Molecular analysis of transcription factors in tomato has suggested probable cross talk between ethylene and abscisic acid (Figure S7). It is proposed that, ABA promotes basal ethylene biosynthesis in system 1 during fruit growth and likely plays a role in ripening regulation after the onset of ripening process [50]. In our analysis, level of abscisic acid was also found to be significantly low during fruit ripening in the spongy tissue, which might be concurrent with lower level of ethylene. Abscisic acid is also known as stress hormone and acts as an antioxidant. Decreasing level of abscisic acid in the spongy tissue justified the prediction of fruit being under stress condition.

Ripening of fruit is correlated with textural changes in the mesocarp. In the spongy tissue reduced ripening affected the textural qualities of the mango fruit. Lignification maintains the firmness of cellular structure. Lignin biosynthesis takes place via phenylpropanoid pathway. This versatile pathway is also involved in phenolics and anthocyanin biosynthesis. In case of the spongy-control tissue mesocarp, 4-O-β-d-glucosyl-4-hydroxycinnamate which is a glucoside derivative of coumaric acid (intermediate in phenylpropanoid pathway) was 4-fold higher at the ripe stage as compared to the mature raw stage of the healthy fruit. Conversely, the concentration was less in the spongy mesocarp at the ripe stage as compared to the other two tissue sets. Concentration of feruloyl glucose was also found to be consistently less in the spongy tissue suggesting defect in lignin biosynthesis. Thus, cumulatively reduced activity of ripening enzymes, altered lignin levels and increased starch accumulation affected the texture of the fruit. Taste is another important characteristic contributing to the flavor of a fruit. Spongy mesocarp is acidic, tangy in nature which might be due to higher accumulation of isocitric acid and amino acids in the mesocarp even at the completely ripe stage as noticed in the present study. During normal ripening of mango, starch is hydrolyzed by amylase into simpler sugars such as glucose, fructose and sucrose in the pulp. Because of heat, activities of these enzymes are reduced, and fine tuning of sugar metabolism is affected ultimately retarding carbohydrate metabolism in the spongy tissue. In case of the healthy and the spongy-control mesocarp, the change in sucrose concentration was up to 10 to 13-folds during ripening as compared to the mature raw stage of the healthy mesocarp whereas, no such significant difference was seen in the spongy mesocarp. Thus, the balance between the sweetness and acidity could not be maintained in the affected spongy tissue part, which hampers the taste of mesocarp and eventually the acceptability and marketability of the fruit.

In conclusion, current study of Alphonso mango fruit using global metabolomic approach has highlighted the correlation between metabolic changes with physiological changes, such as anatomical and organoleptic, in the spongy tissue formation. It has shed light on important and differentially accumulated metabolites and metabolic pathways in this disorder. Though the exact inducing factor of the disorder is difficult to identify, study has given an insight about the metabolite dynamics in spongy, spongy-control and healthy tissues encompassing the entire period of mango fruit ripening. We postulate an abiotic stress induced shift of TCA cycle, GABA shunt and ascorbate glutathione cycle leading to oxidative stress producing spongy tissue malady in Alphonso mango.

## 4. Materials and Methods

### 4.1. Plant Material

Fruits of *Cv.* Alphonso were harvested and collected from mango orchards of Agronomy department of the Dr. Balasaheb Sawant Konkan Agricultural University, Dapoli (N17°45′ E73°11′). Fruits were collected from six individual trees which were considered as biological replicates. Collected fruits were kept in hay containing boxes for ripening. Based on fruit color (redness, yellowness and light intensity) and textural (softness) measurements four ripening stages of Alphonso mango fruit viz. mature raw (90 days after pollination, DAP), table green (5 days after harvest, DAH), mid ripe (10 DAH) and ripe (15 DAH) [51,52], respectively were selected to evaluate the complete ripening period. At each ripening stage, mangoes were removed from hay boxes, and spongy affected mesocarp was separated, frozen in liquid nitrogen and stored at −80 °C until further use. Mesocarp from two fruits of each biological replicate was pulled and considered as a single sample at each ripening stage. Along with the spongy part non-spongy mesocarp around the spongy affected area of the same fruit was also collected separately and considered as spongy-control. Spongy tissue is visually not distinguishable at mature raw stage; hence spongy-control tissue was collected from table green stage onwards. Also, pulp tissue from completely healthy fruits (free from the spongy tissue) at the corresponding ripening stages was also collected and considered as Healthy control in this analysis.

### 4.2. Scanning Electron Microscopy

Thin sections of healthy and spongy mesocarp from table green and ripe stages were fixed on glass cover slip using 5% glutaraldehyde. Tissue fixation was done for 5 h. in dark followed by ethanol washes in increasing gradient (10 to 100%) each for 10 min. For complete dehydration, each tissue piece was kept submerged in 100% ethanol for 10 min, and finally stored overnight in a desiccator. Completely dehydrated tissue section was transferred on aluminium stubs with double sided carbon tape and sputter coated with thin layer of metallic gold under vacuum for enhanced contrast. Visualization was done using scanning electron microscope FEI Quanta 200 3D Model (Thermo Fisher Scientific, Bedford, MA, USA) at 20 kV voltage. Area of starch granules accumulation in each tissue was calculated in mm^2^ using ImageJ software.

### 4.3. Biochemical Analysis of Spongy Mesocarp Mango Tissues

An antioxidant capacity of all the stages of spongy and healthy mesocarp tissue was estimated spectrophotometrically by phosphomolybdenum assay [53]. For sample preparation, 500 mg ground tissue individually from all the stages was dissolved in DMSO. Mixture was sonicated for 5 min in ice bath for homogenization followed by centrifugation at 4 °C for 10 min. For assay, 100 µL of the supernatant was mixed with 1 mL of phosphomolybdenum reaction mixture (28 mM sodium phosphate and 4 mM ammonium molybdate in 0.6 M sulphuric acid) in aluminium foil covered test tube. Incubation was carried out in water bath at 95 °C for 90 min. After cooling to room temperature (~25 °C), the absorbance of the solutions was measured using a UV-visible spectrophotometer at λ_695_ nm against a blank (100 µL DMSO without sample extract); 0.1 to 1.5 mg of Ascorbic acid (Sigma-Aldrich, St. Louis, MO, USA) was used as standard. The antioxidant capacity was reported as ascorbic acid equivalent (AAE) per gram of tissue.

The phenolic content of all the tissues was estimated using the protocol by Slinkard et al. [54]. 500 mg of mesocarp tissue from each stage was suspended in 5 mL of methanol. Mixture was homogenized by 5 min sonication followed by centrifugation at 4 °C for 10 min. For assay, 50 µL of supernatant was mixed with 50 µL of Folin-Ciocalteu Reagent (Sigma-Aldrich, St. Louis, MO, USA) and incubated in dark for 5 min. Then 50 µL of 6% sodium carbonate and 200 µL of distilled water were added to the assay tube. After gentle mixing, test tube was covered in aluminium foil and incubated in dark for 60 min at 37 °C. After incubation, absorbance of the sample was determined using UV-visible spectrophotometer at λ_765_ nm against a blank (50 µL Methanol without sample extract); 0.1 to 1.5 mg of gallic acid (Sigma-Aldrich) was used as a standard. The phenolic content was reported as gallic acid equivalent (GAE) per gram of tissue.

Soluble proteins were extracted from 1 g mesocarp tissue each of all the stages. Tissue was crushed in liquid nitrogen using mortar and pestle and was mixed with 5 mL of extraction buffer (50 mM Phosphate buffer, pH 7.4, 5 mM MgCl_2_, 2% (*v/v*) glycerol and 2% (*w/v*) polyvinylpyrrolidone). Homogenate was centrifuged at 12,600× *g* for 30 min at 4 °C. The supernatant obtained was used for total protein estimation in microgram of protein per gram of tissue by Bradford method using 0.1 mg to 1.5 mg of BSA (Promega Corporation, Madison, WI, USA) as standard, and enzyme activity assays.

### 4.4. Enzyme Assays

Spectroscopic assays for the activity of three stress-related enzymes—ascorbate peroxidase, dehydroascorbate reductase and catalase—were performed in all the tissue sets as reported earlier [55]. 1 g of tissue was taken for crude protein extraction which was further used to assay the enzyme activities. Average enzyme activities were reported as units/min/gram of tissue.

### 4.5. Metabolite Extraction and UHPLC-Q-Exactive-Orbitrap Mass Spectrometer Analysis

Metabolite analysis was carried out using solvent extraction method, wherein 500 mg fruit pulp of each tissue was crushed in liquid nitrogen using prechilled mortar and pestle. Finely ground tissue was added in 1 mL of 90% ice cold methanol with 0.1% formic acid followed by three cycles of 10 min sonication. Extract was centrifuged at 4 °C at 12,600× *g* for 20 min and supernatant was collected in fresh microcentrifuge tube. To the remaining pellet, 1 mL of 100% ice cold methanol with 0.1% formic acid was added and the whole protocol was repeated. Both the supernatants were pulled and filtered through 0.2 µm Amicon filter (Merck Millipore, Darmstadt, Germany). Filtered supernatant was stored at −80 °C till further use. For the non-targeted analysis of metabolites 1.5 µL of the sample was injected on an Accela™ ultra-high-performance liquid chromatography (UHPLC) system (Thermo Fisher Scientific, Bedford, MA, USA), coupled online via heated electrospray ionization source (HESI) with a Q-Exactive-Orbitrap mass spectrometer (Thermo Fisher Scientific). Chromatographic separation was carried out on Accucore C18 column (2.6 μm × 4.6 mm × 150 mm) (Thermo Fisher Scientific). Temperature of the column was maintained at 40 °C and sample manager temperature was 4 °C. Samples were run on ESI (+) as well as ESI (−) modes. Water (eluent A): Acetonitrile (eluent B) linear gradient system was employed over 15 min with flow rate 400 µL/min. Gradient program was as follows, 0 to 1 min 98% (A)/ 2% (B); 1 to 7 min 70% (A)/ 30% (B); 7 to 9 min 55% (A)/ 45% (B) and 9 to 11 min 30% (A)/ 70% (B). Concentration of eluent B was further increased to 98% till the completion of 13 min. Subsequently, eluent B was returned to 2% in 13.3 min and held for an additional 1.2 min before the injection of next sample. Mass scan range was 100–1000 *m/z* and orbitrap resolution was set at 70,000. In both, the positive and the negative mode, capillary temperature was maintained at 320 °C while sheath gas and auxiliary gas flow rate were maintained at 55 and 12 arbitrary units, respectively. Spray voltage and auxiliary gas heater temperature were 4.2 kV and 350 °C in positive mode while 3.6 kV and 300 °C in negative mode, respectively. The MS/MS was performed in data dependant mode with collision energy between 35–40 eV using nitrogen as collision gas.

### 4.6. Data Analysis

Quality of metabolite profiling data and instrument stability was checked using pooled QC sample prepared by mixing an equal amount extract from all the tissue sets. Five QC runs were performed before analyzing the experimental dataset. For checking the stability during the acquisition of experimental set, QC sample was run after every six samples. Extracted ion chromatograms of Leucine and Isoleucine were used for validation of spectrometric parameters. Raw data conversion from profile mode to centroid mode was carried out using ProteoWizard v3.0 tool [24] for ESI (+) and ESI (−) mode separately and combined later. Data alignment, peak picking and retention time correction were done using customized R based ProMetab tool. The entire procedure was followed as reported earlier [56]. ProMetab equipped with CAMERA, mzMatch, Comb2+ algorithm and metExplore packages helps in processing and identification of features along with converting the data to a format for multivariate analysis. Multivariate statistical analysis was carried out using web-based tool MetaboAnalyst 4.0 (http://www.metaboanalyst.ca) [57,58,59]. Principle component analysis (PCA) was done using mean centered data to analyse the difference between various tissue types and pareto scaling was used for PLS-DA and OPLS-DA analysis for highlighting their biological significance. From all the combinations of healthy, spongy-control and spongy tissue at four different stages, VIP values from 7-fold cross validated OPLS-DA model for all the peaks were identified. VIP score greater than or equal to 2 was selected suggesting that these variables had the highest influence in discriminating healthy and spongy tissue. Identification of shortlisted metabolite features were performed using exact mass by KEGG (https://www.genome.jp/kegg/) and METLIN (https://metlin.scripps.edu/landing_page.php) databases. Metabolite features were tentatively annotated at level 2 of metabolomics standards initiative (MSI) [20]. Validation of these metabolites was carried out by tandem mass spectrometry. Mass fragmentation of candidate metabolite was obtained using the Mass Frontier^TM^ (Thermo Fisher Scientific) software. Fragmentation pattern of candidate metabolite after tandem spectroscopy was compared with known mass fragmentation pattern using in house R script. Minimum six spectral matches within known fragmentation pattern and tandem mass fragmentation pattern or ppm error (within mass with adduct and probable mass) less than five were set as threshold for confirmation of a metabolite.

### 4.7. Statistical Analysis

For microscopic analysis, two images for each tissue set were scanned and the area of starch accumulation was calculated from the average values of five different regions of each image. Image normalization n was done by the scale represented on each image. Biochemical and enzyme assays were performed separately for each tissue set considering six biological replicates and each assay was repeated thrice for statistical significance. Univariate analysis of the peak intensities of the putative metabolites was done using six biological replicates. Fischer’s LSD test (*p* ≤ 0.05) was carried out after ANOVA (StatView software, version 5.0, SAS Institute Inc., Cary, NC, USA) on healthy, spongy-control and spongy tissue separately to compare the quantity of each compound within three datasets of four ripening stages.

## Figures and Tables

**Table 1:** List of putative metabolites identified and validated by tandem mass spectrometry. Metabolites annotated at the level 2 of Metabolites Standards Initiatives (MSI) [20]. Putative metabolites are arranged with the ascending order of exact masses (identified through KEGG database).

Putative Metabolite	Formula	KEGG	No. of Fragment Matches	RT (min)	Probable Mass	Exact Mass	Adduct Annotation	ESI Mode
1-Aminocyclopropane-1-carboxylic acid	C_6_H_7_NO_2_	C01234	5	3.02	101.0481	101.0477	[M+H]^+^	Positive
γ-Aminobutyric acid ^a^	C_4_H_9_NO_2_	C00334	6	3.02	103.0638	103.0633	[M+H]^+^	Positive
Uracil	C_4_H_4_N_2_O_2_	C00106	4	5.06	112.0276	112.0273	[M+H]^+^	Positive
l-Proline	C_5_H_9_NO_2_	C00148	11	3.12	115.0636	115.0633	[M+H]^+^	Positive
l-Threonine	C_4_H_9_NO_3_	C00188	8	3	119.0584	119.0582	[M+H]^+^	Positive
Pyroglutamate	C_5_H_7_NO_3_	C01879	15	4.62	129.0426	129.0426	[M+H]^+^	Positive
Aspartate	C_4_H_7_NO_4_	C00049	6	4.6	133.036	133.0375	-	Positive
Spermidine	C_7_H_19_N_3_	C00315	9	2.82	145.1579	145.1579	[M+H]^+^	Positive
α-Ketoglutaric acid	C_5_H_6_O_5_	C00026	7	4.61	146.0215	146.0215	[M+H]^+^	Positive
l-Lysine	C_6_H_14_N_2_O_2_	C00047	8	2.81	146.1055	146.1055	[M+H]^+^	Positive
(*S*)-2-Hydroxyglutarate	C_5_H_8_O_5_	C03196	10	4.59	148.036	148.0372	[M-H]^−^	Negative
Methionine	C_5_H_11_NO_2_S	C00073	9	4.13	149.051	149.051	[M+H]^+^	Positive
Histidine	C_6_H_9_N_3_O_2_	C00135	12	2.83	155.0695	155.0695	[M+H]^+^	Positive
DL-2-Aminoadipate	C_6_H_11_NO_4_	C00956	24	3.4	161.0688	161.0688	[M+H]^+^	Positive
2-Hydroxyadipate	C_6_H_10_O_5_	C02360	25	3.14	162.0527	162.0528	[M+H]^+^	Positive
Phenylalanine	C_9_H_11_NO_2_	C00079	20	6.77	165.079	165.079	[M+H]^+^	Positive
Dehydroascorbic acid	C_6_H_6_O_6_	C05422	7	4.02	174.0161	174.0164	[M+H]^+^	Positive
l-Arginine	C_6_H_14_N_4_O_2_	C00062	19	2.89	174.1111	174.1117	[M+H]^+^	Positive
Ascorbic acid	C_6_H_8_O_6_	C00072	24	3.7	176.0321	176.0321	[M+H]^+^	Positive
D-Glucose	C_6_H_12_O_6_	C00031	11	3.69	180.0624	180.0634	[M+H]^+^	Positive
Tyrosine	C_9_H_11_NO_3_	C00082	17	3.38	181.0734	181.0739	[M+H]^+^	Positive
Isocitric acid	C_6_H_8_O_7_	C00311	16	3.66	192.0267	192.027	[M-H]^−^	Negative
ferulic acid	C_10_H_10_O_4_	C01494	5	7.78	194.0583	194.0579	[M+H]^+^	Positive
N2-Acetyl-l-aminoadipate	C_18_H_13_NO_5_	C12986	35	3.06	203.0792	203.0794	[M+H]^+^	Positive
l-Tryptophan	C_11_H_12_N_2_O_2_	C00078	10	7.95	204.0899	204.0899	[M+H]^+^	Positive
Indole acetic acid	C_11_H_11_NO_3_	C02043	6	10.1	205.0734	205.0739	[M+H]^+^	Positive
Sedoheptulose	C_7_H_14_O_7_	C02076	19	3.23	210.0737	210.074	-	Positive
Succinylproline	C_9_H_13_NO_5_	C11711	35	3.22	215.0788	215.0794	[M+H]^+^	Positive
Pyridoxal Phosphate	C_8_H_10_NO_6_P	C00018	1	4.62	247.0245	247.0246	[M+H]^+^	Positive
(+)-Abscisic Acid	C_15_H_20_O_4_	C06082	18	12.69	264.1362	264.1362	[M-H]^−^	Negative
Deidaclin	C_12_H_17_NO_6_	C08329	34	3.1	271.1032	271.1056	[M+H]^+^	Positive
Ranunculin	C_11_H_16_O_8_	C08512	19	3.08	276.082	276.0845	[M+H]^+^	Positive
Kaempferol	C_15_H_10_O_6_	C05903	2	6.24	286.0479	286.0477	[M+H]^+^	Positive
Sphinganine	C_18_H_39_NO_2_	C00836	8	14.45	301.2979	301.2981	[M+H]^+^	Positive
Glutathione reduced	C_10_H_17_N_3_O_6_S	C00051	22	4.72	307.0813	307.0838	[M+H]^+^	Positive
Phytosphingosine	C_18_H_39_NO_3_	C12144	18	14.19	317.2929	317.293	[M+H]^+^	Positive
4-O-β-d-glucosyl-4-hydroxycinnamate	C_15_H_18_O_8_	C04415	11	7.38	326.1002	326.1002	[M+2Na-H]^+^	Negative
9,10-Dihydroxy-12,13-epoxyoctadecanoic acid	C_18_H_34_O_5_	C14837	11	13.3	330.2408	330.2406	[M-H]^-^	Negative
5′-O-β-d-glucosyl-pyridoxine	C_14_H_21_NO_8_	C03996	13	4.48	331.1265	331.1267	[M+H]^+^	Positive
Sucrose	C_12_H_22_O_11_	C00089	17	3.82	342.1162	342.1162	[M-H]^−^	Negative
Chlorogenic acid #	C_16_H_18_O_9_	C00852	15	3.45	354.09	354.0951	[M-H]^−^	Negative
1-Feruloyl-d-glucose	C_16_H_20_O_9_	C17759	10	8.99	356.1092	356.1107	[M-H]^−^	Negative
3-O-Feruloylquinic acid	C_17_H_20_O_9_	C02572	14	3.08	368.1123	368.1107	[M-H]^−^	Negative
Vernoflexuoside	C_21_H_28_O_8_	C09579	6	9.07	408.176	408.1784	[M+H]^+^	Positive
Mangiferin	C_19_H_18_O_11_	C10077	14	8.61	422.0848	422.0849	[M+H]^+^	Positive
Glutathione oxidized	C_20_H_32_N_6_O_12_S_2_	C00127	14	5.16	612.1518	612.152	[M+2H]^+^	Positive

^a^ Compound validated using Metlin score. # Compounds validated with the fragment match of standard compounds as well.

**Table 2:** Fold change variation in the putative metabolite composition within healthy, spongy and spongy control mesocarp tissue of mango. Fold change was calculated by considering the values of mature raw healthy stage as 1.

Metabolite	Fold Change in Healthy Tissue			Fold Change in Spongy Control Tissue			Fold Change in Spongy Tissue			
	Table Green	Mid Ripe	Ripe	Table Green	Mid Ripe	Ripe	Mature Raw	Table Green	Mid Ripe	Ripe
1-Aminocyclopropane-1-carboxylic acid	1.6	1.17	1.86	0.53	1.13	2.22	0.91	0.47	0.31	0.63
γ-Aminobutyric acid	1.03	0.19	0.35	0.41	0.27	0.27	1.53	2.28	0.72	1.03
Uracil	5.06	4.23	10	4.43	11.5	18.4	0.91	47.63	14.08	16.49
l-Proline	1.34	0.29	0.34	0.29	0.25	0.4	1.54	0.86	0.4	0.88
l-Threonine	1.72	0.58	1.32	0.56	0.61	1.06	1.41	1.21	0.68	1.23
Pyroglutamate	1.05	0.22	0.23	0.2	0.21	0.27	1.16	5.38	5.35	4.69
Aspartate	1.43	0.78	0.8	0.34	0.45	0.49	0.84	0.11	0.18	0.19
Spermidine	1.43	0.12	0.83	0.07	0.28	0.52	0.76	0.32	0.1	0.17
α-Ketoglutarate	0.98	0.13	0.1	0.17	0.06	0.07	1.01	0.72	0.48	0.62
l-Lysine	0.78	0.18	0.22	0.2	0.19	0.25	0.87	1.39	0.62	0.64
2-Hydroxyglutarate	2.15	0.65	0.58	0.95	0.36	0.75	1.45	1.65	1.81	2.81
Methionine	0.73	0.24	0.25	0.36	0.32	0.44	0.8	1.24	0.38	0.43
Histidine	1.57	1.29	5.44	0.57	1.6	3.36	1.6	1.43	0.77	1.18
DL-2-Aminoadipate	1.31	0.19	0.44	0.08	0.21	0.33	1.55	0.31	0.14	0.22
2-Hydroxyadipic acid	2.4	2.66	4.62	3.41	3.48	3.96	0.9	1.83	2.25	1.65
Phenylalanine	0.51	0.07	0.04	0.06	0.04	0.04	1.08	0.8	0.22	0.31
Dehydroascorbic acid	2.04	2.26	2.79	2.28	1.65	2.33	1.28	3.47	1.9	3.58
l-Arginine	0.98	0.02	0	0.03	0.01	0.01	1.11	0.87	0.28	0.17
Ascorbic acid	0.73	0.22	1.31	0.63	0.28	1.62	1.01	0.14	0.05	0.23
D-Glucose	1.07	0.44	0.67	0.48	2.22	0.69	0.83	4.4	5.42	1.24
Tyrosine	1.51	0.4	0.86	0.73	0.66	1.47	1.32	2.47	1.92	2.65
Isocitric acid	2.69	0.48	0.31	0.84	0.25	0.3	1.66	1.87	1.81	1.94
ferulic acid	1.85	1.2	2.53	1.55	1.98	3.44	1.22	2.05	2.28	3.98
N2-Acetyl-L-aminoadipate	1.85	1.36	3.19	1.95	2.4	4.53	0.94	2.52	2.6	4.26
l-Tryptophan	1.81	0.43	0.84	0.39	0.52	1.47	1.37	2.33	0.9	1.81
Indole acetic acid	1.55	0.37	0.59	0.4	0.47	1.75	1.28	2.39	0.87	2.07
Sedoheptulose	1.51	1.87	5.05	4.11	6.59	14.31	1.76	1.03	2.29	3.32
Succinylproline	1.54	0.59	1.39	0.91	1.44	2.01	1.13	1.89	1.32	2.34
Pyridoxal Phosphate	0.99	0.43	0.45	0.53	0.27	0.38	1.04	0.98	0.84	1.45
(+)-Abscisic Acid	4.03	4.12	2.83	2.23	1.54	2.1	1.13	1.21	0.44	0.35
Deidaclin	1.87	0.59	1.53	1.86	2.74	4.53	1.02	1.09	7.69	90.34
Ranunculin	0.97	0.86	1.7	1.47	0.76	1.71	0.91	1.54	1.59	3.34
Kaempherol	1.05	0.65	1.5	0.47	0.82	1.47	0.88	0.28	0.46	1.89
Sphinganine	1.23	0.46	0.01	0.16	0.21	0.01	0.21	0.21	0.22	0.01
Glutathione reduced	1.99	0.31	0.44	0.52	0.32	0.34	1.11	0.23	0.03	0.05
Phytosphingosine	1.08	0.35	0.12	0.47	0.26	0.1	1.13	1.48	1.25	0.3
4-O-β-d-glucosyl-4-hydroxycinnamate	1.27	1.36	2.97	1.65	1.26	4.24	1.33	1.32	1.5	2.22
9,10-Dihydroxy-12,13-epoxyoctadecanoic acid	1.07	0.57	0.69	1.16	0.83	2.01	1.01	3.37	35.87	132.76
5′-O-β-D-glucosyl-pyridoxine	2.08	2.8	6.8	6.13	7.86	12.65	1.18	7.51	27.24	92.21
Sucrose	3.8	8.23	9.38	5.65	11.74	13.56	0.84	3.54	2.47	1.44
Chlorogenic acid	0.8	1.14	10.8	1.8	1.26	15.04	1.22	1.47	2.68	19.97
1-Feruloyl-d-glucose	0.81	0.41	0.01	0.45	0.23	0.01	0.72	0.01	0.02	0.04
3-O-Feruloylquinate	1.15	1.67	6.8	1.75	0.8	4.55	1.5	0.32	0.74	5.51
Vernoflexuoside	2.76	3.98	20.19	2.67	4.58	14.42	1.47	5.72	4.45	24.35
Mangiferin	0.56	0.37	0.57	0.32	0.43	1.22	0.44	1.06	1.84	1.3
Glutathione oxidized	1.56	1.33	0.19	0.24	0.44	0.27	1.86	4.91	1.76	0.67

## Supplementary Materials

The following are available online at https://www.mdpi.com/2218-1989/9/11/255/s1, Figure S1: Overlaid chromatograms of Leucine and Isoleucine among the QC runs, Figure S2: Workflow for LC-MS data, Figure S3: Tissue specific metabolite variation, Figure S4: Two-way ANOVA for the effect of phenotype, stage of ripening and their interaction, Figure S5a: Bar graphs of differentially accumulated amino acids, sugars, hormones and metabolites involved in adipic and shikimic acid pathway along with sphinganine biosynthesis in healthy, spongy control and spongy tissue within four stages of ripening (mature raw, table green, mid ripe and ripe) in Alphonso mango, Figure S5b: Bar graphs of differentially accumulated phenolics and metabolites involved in shikimic acid pathway in healthy, spongy control and spongy tissue within four stages of ripening (mature raw, table green, mid ripe and ripe) in Alphonso mango. Y-axis denotes the peak intensity values. Figure S6: Physiological changes occurring in mesocarp during mango fruit development, Figure S7: Crosstalk between ethylene and abscisic acid in fruit ripening Table S1: Peak intensities of the extracted ions from UHPLC-Q-Exactive-Orbitrap analysis from all the three tissue sets (healthy, spongy control, spongy) at four ripening stages (mature raw, table green, mid ripe, ripe) of alphonso mango mesocarp including all six biological replicates.
