# Supplementary figures and images for: Metabolomic Dynamics Reveals Oxidative Stress in Spongy Tissue Disorder During Ripening of Mangifera indica L. Fruit
Source: Metabolites. 2019 Oct 29;9(11):255. doi: 10.3390/metabo9110255 (PMC6918312; doi:10.3390/metabo9110255)

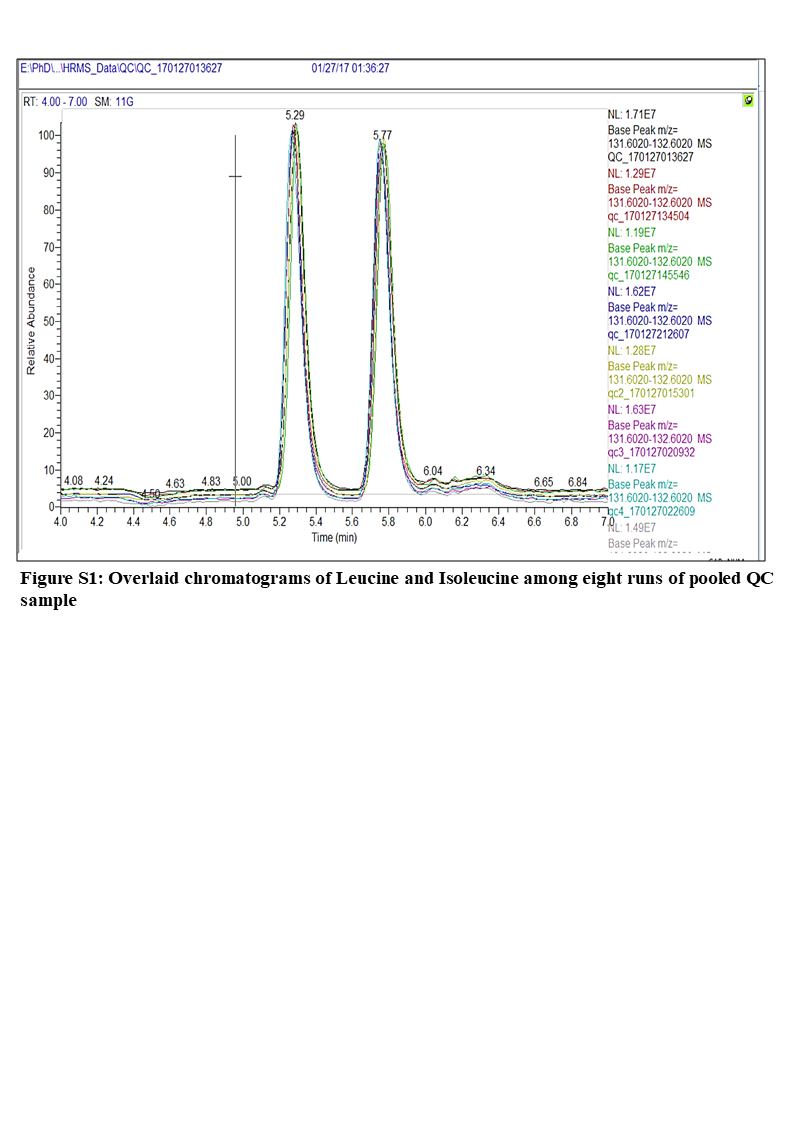

Supplement: Supplementary file 1 [file metabolites-09-00255-s001.zip › Figure S1.tif]

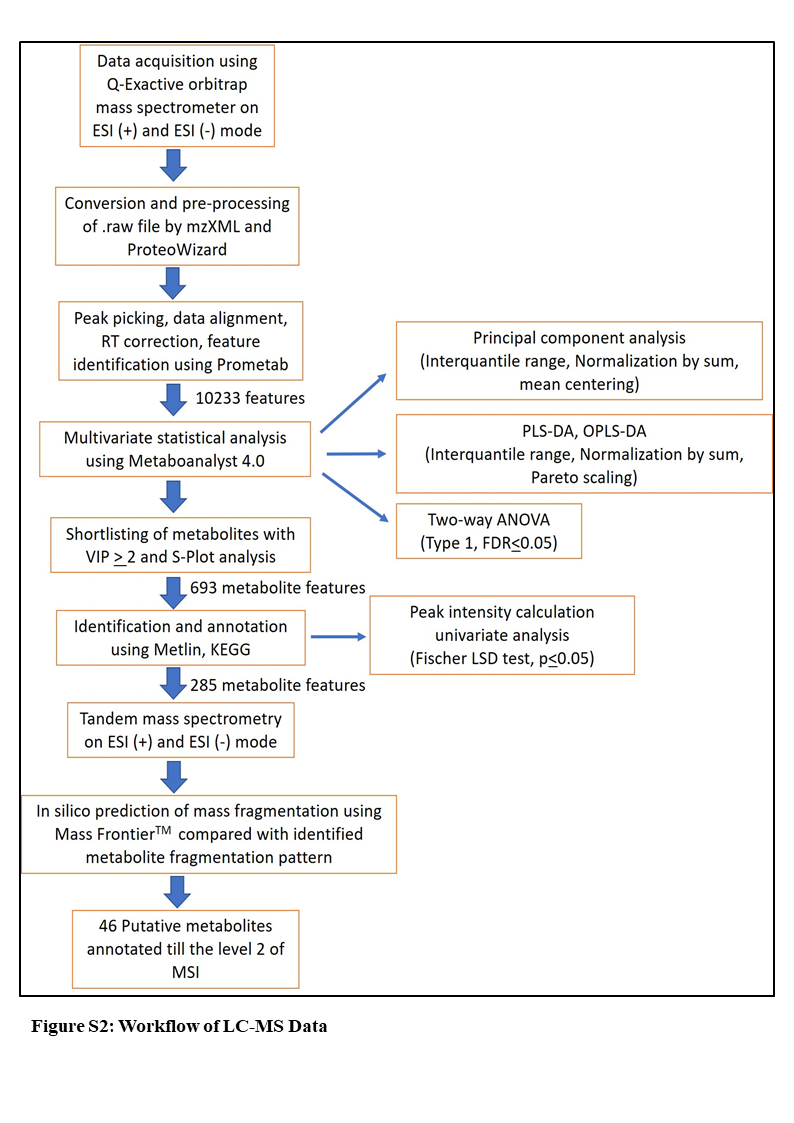

Supplement: Supplementary file 1 [file metabolites-09-00255-s001.zip › Figure S2.tif]

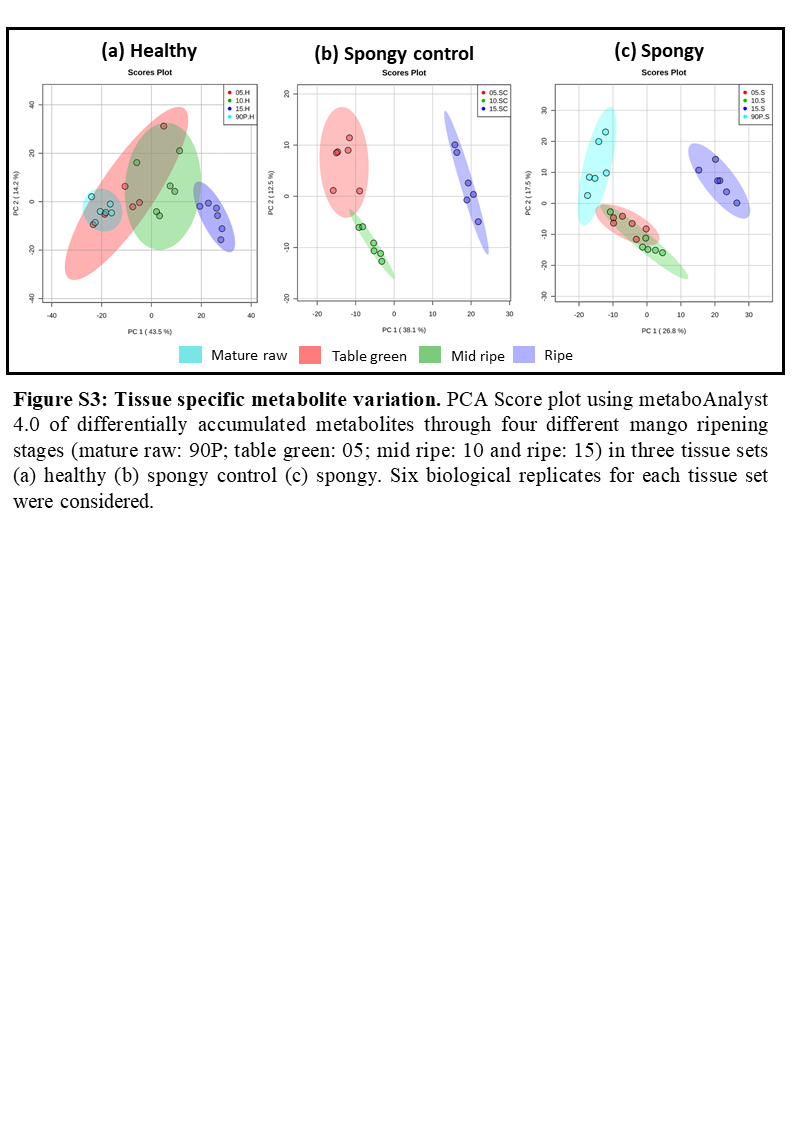

Supplement: Supplementary file 1 [file metabolites-09-00255-s001.zip › Figure S3.tif]

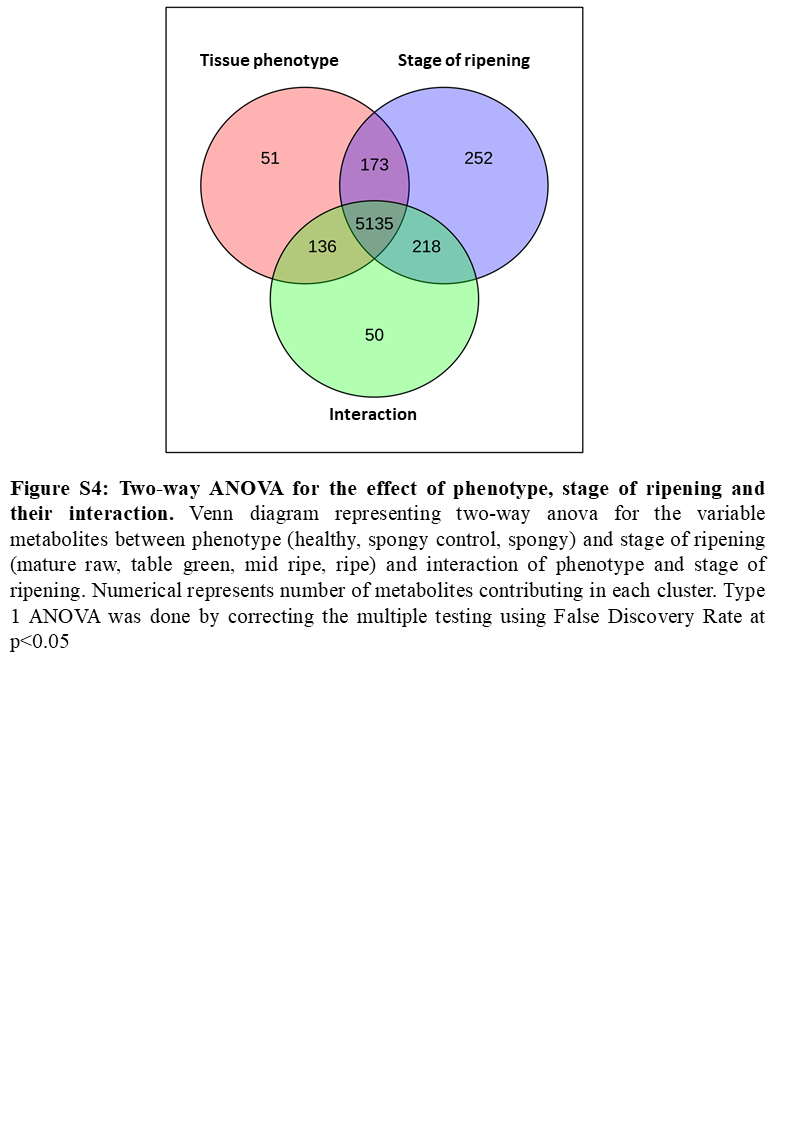

Supplement: Supplementary file 1 [file metabolites-09-00255-s001.zip › Figure S4.tif]

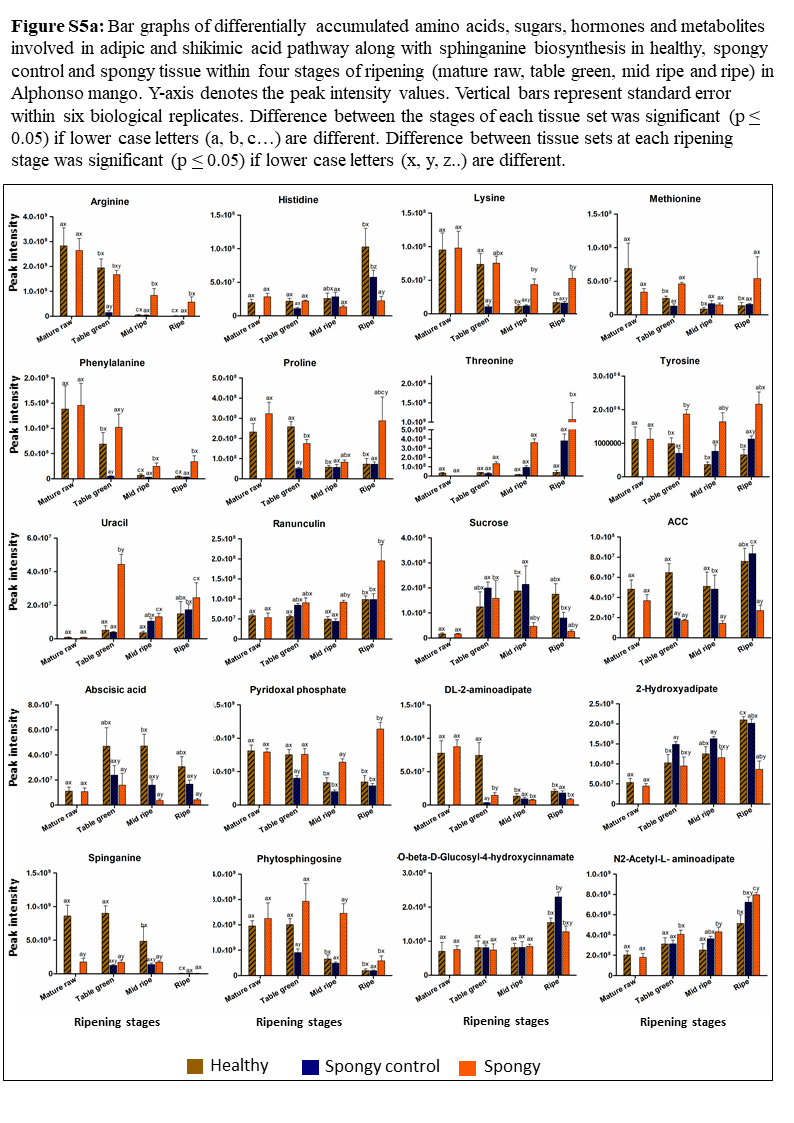

Supplement: Supplementary file 1 [file metabolites-09-00255-s001.zip › Figure S5a.tif]

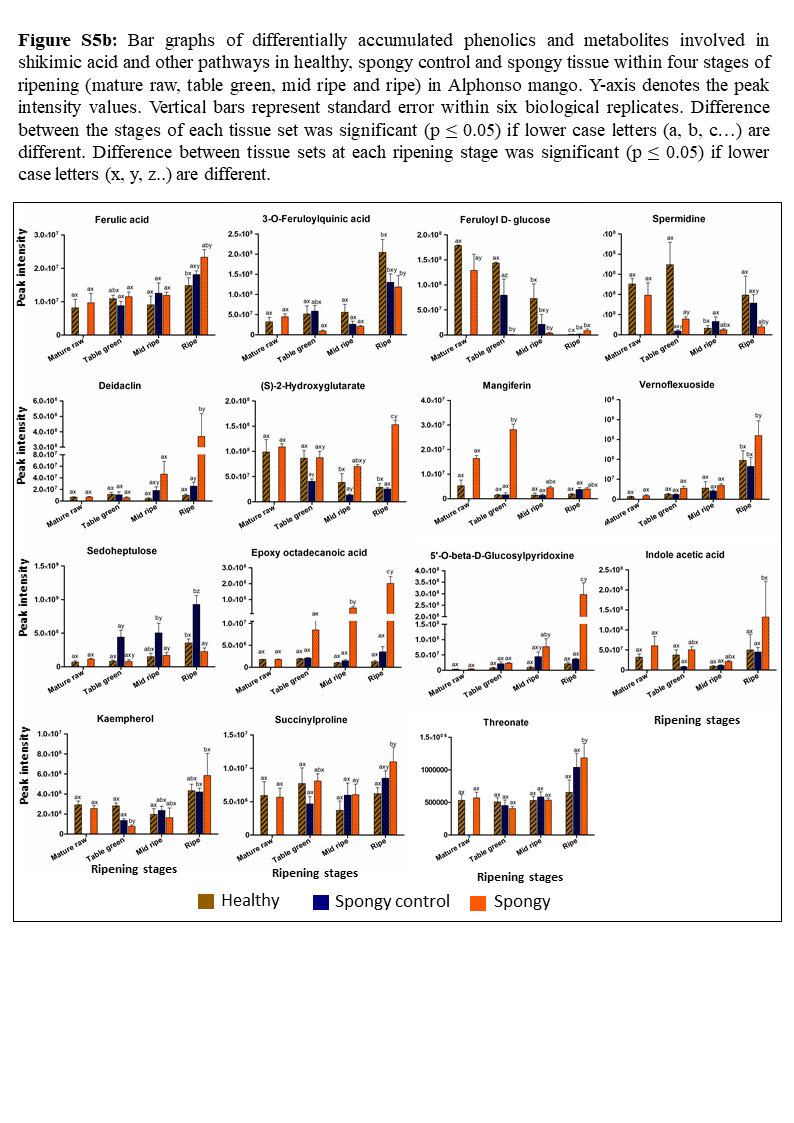

Supplement: Supplementary file 1 [file metabolites-09-00255-s001.zip › Figure S5b.tif]

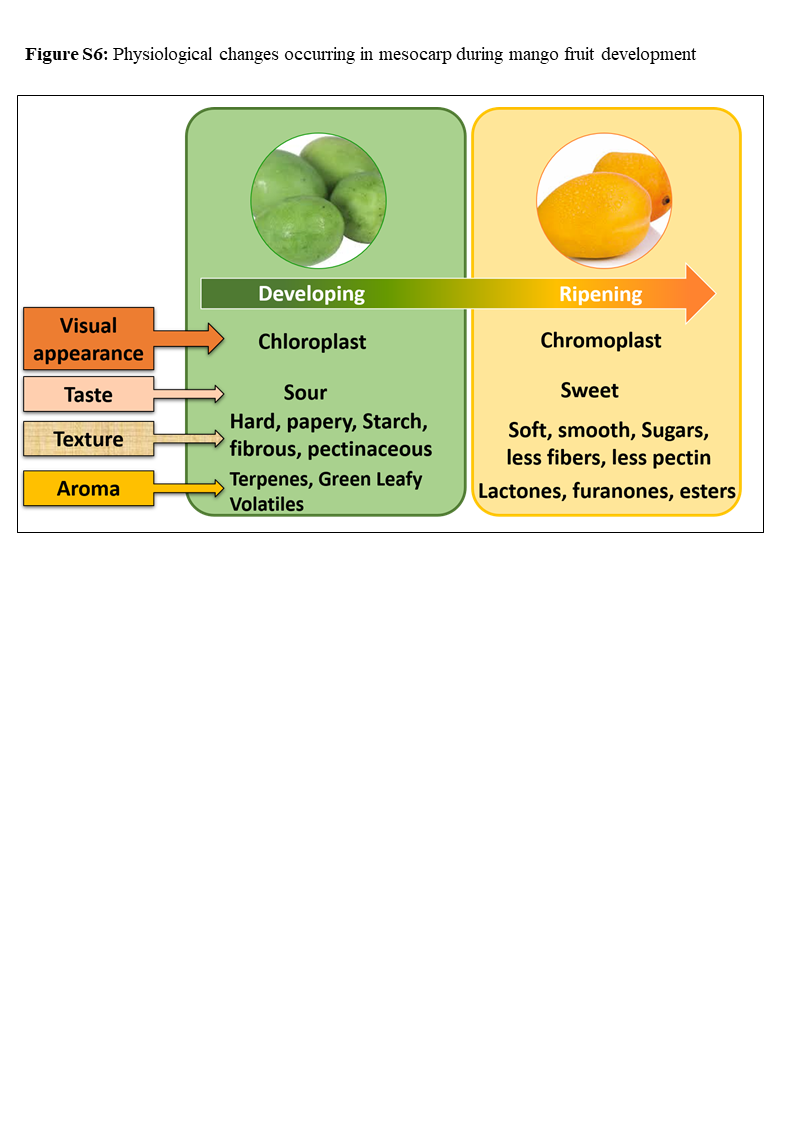

Supplement: Supplementary file 1 [file metabolites-09-00255-s001.zip › Figure S6.tif]

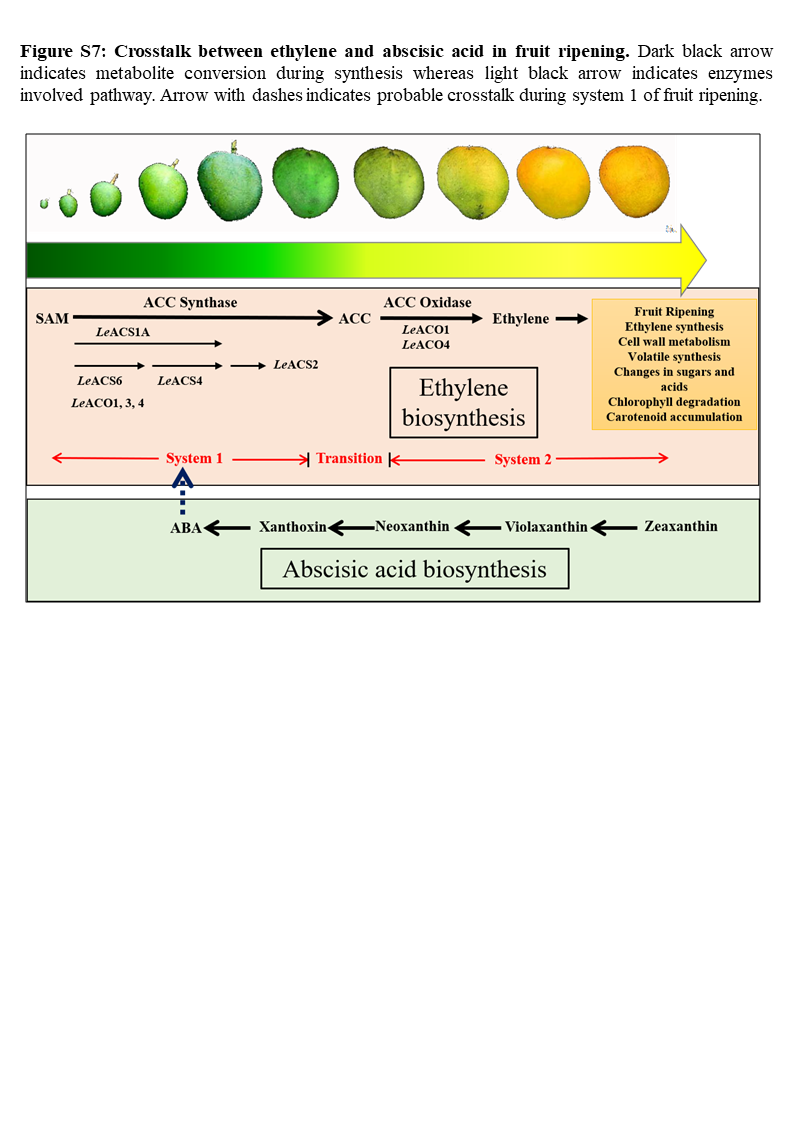

Supplement: Supplementary file 1 [file metabolites-09-00255-s001.zip › Figure S7.tif]
